# Facing the struggle alone; Insights into the experience of PhD scholars in Pakistan

**DOI:** 10.12669/pjms.41.7.11414

**Published:** 2025-07

**Authors:** Naheed Mahsood, Usman Mahboob, Naveed Afzal Khan

**Affiliations:** 1 Naheed Mahsood Department of Medical Education, Khyber Girls Medical College, Peshawar, Pakistan; 2 Usman Mahboob Institute of Health Professions Education, Khyber Medical University, Peshawar, Pakistan; 3 Naveed Afzal Khan Department of Medical Education, Khyber Girls Medical College, Peshawar, Pakistan

**Keywords:** Challenges, Doctorate, Enablers, Medical education, Medical university, PhD, Scholar, Supervisor

## Abstract

**Objective:**

Explore the key challenges encountered by PhD scholars in Pakistan and the coping strategies they employ to address these challenges.

**Method:**

A qualitative phenomenological approach with purposive sampling was used to conduct six in-depth interviews with PhD scholars and five in-depth interviews with the supervisors. Guiding questions were searched from the literature, authenticated by three experts in the Medical Education Department of Khyber Medical University, and piloted before implementing the study. The duration of the study was two years. Data was recorded verbatim using a Dictaphone. Transcription was done through transcriber software. Thematic analysis was performed.

**Results:**

Four themes were extracted, namely: Always alone in the struggle (subtheme: experience), Progress, not perfection (subtheme: enablers), confined by the invisible walls (subtheme: inhibitors/barriers), and the Key to Success (subtheme: recommendations).

**Conclusion:**

PhD is a lonely journey with many ups and downs during its progression. Enablers contain essential skills, dedication, and a robust support system. Socioeconomic status, marital status, and financial limits strongly influence this journey. Recommendations include financial support, workshops, encouraging self-reliance, and excellent time management. Understanding these factors provides a framework for establishing policies to enhance PhD scholars’ success in the unique setting of developing nations like Pakistan.

## INTRODUCTION

The volume of doctoral education in medical universities in developing countries is increasing significantly.^1^ PhD candidates typically encounter stress, uncertainty, and problems within the new academic environment during their doctoral studies, which can impact their academic development.^2^ The high attrition rate among PhD scholars has been documented in various countries and disciplines.^3^ The extended duration and increased intensity of doctoral programs result in elevated stress levels for candidates. PhD candidates occasionally encounter increased ambiguity as a result of the open-ended nature of their research activities and the aspiration to make a unique contribution to their field.^4^ Doctoral research is more intellectually and methodologically challenging than master’s or undergraduate work due to its breadth and complexity.^5^

Doctoral candidates frequently experience isolation as a result of the autonomous nature of their research, resulting in psychological challenges.^6^ Doctoral programs include navigating significant uncertainty, encompassing ambiguity in research objectives, methodologies, and results. Doctoral programs typically provide less structured instruction and guidance compared to master’s or undergraduate degrees, necessitating increased self-direction.^7^ PhD candidates frequently encounter difficulties in obtaining sufficient funding for their study, resulting in financial strain and resource limitations.^8^

In developing countries, universities may still be evolving in terms of infrastructure, research facilities, and academic resources,^9^ providing hurdles for PhD candidates. Candidates in developing countries often face severe constraints in accessing research funds. This leads to challenges in accessing pertinent resources thus limiting the scope and depth of their projects.^10^ Unlike in developed countries where established training for supervisors is frequent, supervisors in developing countries may lack standardized training, relying on trial and error.^11^

Accordingly, there is a need to develop an in-depth understanding of issues and problems PhD scholars face during their doctoral journey and how these problems can affect the successful completion of this journey, the dark corners and hidden mysteries of this journey, and the coping strategies. The focus of this study was to explore the challenges PhD scholars face in Pakistan and how they cope with these problems.

## METHODS

Qualitative study design and phenomenology approach were used to explore the experience of PhD scholars and PhD supervisors and the meaning they attribute to their experience. The duration of the study was two years. Purposive sampling was done, and six in-depth interviews were conducted with the scholars, along with five in-depth interviews with the supervisors in basic medical sciences at different medical universities in Pakistan till saturation of data was achieved. The interviews were conducted via a hybrid methodology tailored to the participants’ preferences and convenience. We performed two interviews with PhD scholars via Zoom and four in person, while three interviews with supervisors were conducted via Zoom and two in person. This flexible approach facilitated efficient data gathering by considering participant availability and location.

### Ethical Approval:

The study was granted ethical approval via notification No. DIR/KMU-EB/JP/000862; Dated: July 20, 2021 by the university ethics committee of Khyber Medical University.

All those PhD scholars in basic medical science who were at the thesis stage and had completed their coursework were included in the study. The PhD supervisors whose scholars completed their PhD or at least their coursework were included in the study. Guiding questions were searched from the literature and authenticated by three experts in the Medical Education Department of Khyber Medical University. They were piloted before implementing the study. Data was recorded using a Dictaphone. Transcription was done through transcriber software. After each session, the moderator and assistant moderator reviewed all the transcripts and coded the data under various headings. Thematic analysis was performed. Several techniques were adopted to ensure the quality of the qualitative research. Piloting and member checking ensured credibility. Triangulation was adopted in the data analysis to enhance confirmability.

## RESULTS

**Fig.1:**
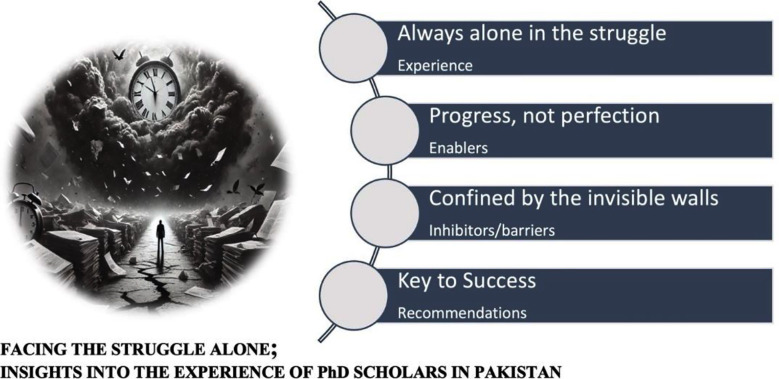
Themes identified by the participants.

The 1st cycle of coding of the analysis was done by open coding. The 2nd coding cycle was done to find out the relationships by axial coding. The thematic analysis was done to create meaningful patterns. The results were summarised based on thematic categorisations. A total of 93 codes (where 39 were in PhD scholar’s interviews and 54 in PhD supervisors’ interviews) were identified in the first cycle of coding, which further led to 40 codes (where 16 were in PhD scholar’s interviews and 24 in PhD supervisors’ interviews) in the 2nd cycle of coding. Finally, four themes were extracted, namely: Always alone in the struggle (subtheme: experience), Progress, not perfection (subtheme: enablers), confined by the invisible walls (subtheme: inhibitors/barriers), and the Key to Success (subtheme: recommendations) shown as Fig.1.

### Always alone in the struggle: Experience:

All participants in the in-depth interviews, including scholars and supervisors, unanimously acknowledged the immense challenges and hardships faced by PhD scholars. They emphasised that these struggles are typically faced alone. The majority of participants expressed the belief that marital status, socioeconomic status, and age have a significant impact on a scholar’s journey. Specifically, married scholars have additional responsibilities towards their spouse and children, while scholars with a favourable socioeconomic status find their journey facilitated. Furthermore, advancing age adds further difficulty to the scholar’s journey. Additionally, they determined that if scholars stay in the same location as the institution, it greatly simplifies their travel, saving them time, energy, and transportation expenses. However, the majority of participants stated that their race had no impact on their journey as PhD scholars. The corresponding codes and representative quotes are outlined in Table-I.

**Table-I:** Themes & subthemes with their corresponding codes and representative quotes.

#	Themes	Subthemes	Codes	Representative Quotes
1	Always alone in the struggle: Experience	Scholar’s Personal Journey Environmental Influences Supervisory Perspectives	Every day is challenging in this journey. Effects of Marital Status and Gender on this Journey Effects of Age of Scholar Effects of Residence of Scholar Effects of Socioeconomic Status of the Scholar Experience of a supervisor with scholars	"I felt very isolated, it seemed that I faced all obstacles of my PhD alone without any help, from course work to comprehensive exams, from synopsis development to the writing of papers, I felt lonely and vulnerable.” Participant D “The route of PhD scholars is complex, and many factors affect their experience as doctoral candidates e.g. age, gender, marital status, socioeconomic status, and distance of residence from the university. Supervisors assist academics in navigating this complex terrain, where the distinct experiences of each individual contribute to a narrative that encompasses the varied aspects of scholarly research.” Participant B
2	Progress, not perfection Enablers	Academic and Research Skills Social and Collaborative Support Institutional and Systemic Support	Skills Practice for academic writing Time management Dedication and punctuality Peer support Self-management skills of scholars Ethical and Research Boards Support from supervisor Support from university	“As PhD scholars explore the complex terrain, refining their abilities, seeking comfort in the support of their peers, and depending on the strong foundation of institutional support, the combined experience serves as evidence of the harmonious relationship between individual expertise and a helpful academic environment.” *Participant A*
3	Confined by the invisible walls. Inhibitors/barriers	Supervisor and Scholar Dynamics Resource and Curriculum Challenges Time and Workload Pressures:	Lack of support from the supervisor Lack of feedback from the supervisor Lack of dedication of scholar Lack of punctuality of scholar Financial constraints Improper sequence of curriculum Workload and time constraints of supervisors Lack of resources The casual attitude of scholars toward research The capacity of supervisors	"Doctoral students usually face challenges such as the lack of support and guidance from supervisors, financial limitations, and a curriculum that is often not well-matched." Participant E
4	Key to Success Recommendations	Scholarly Empowerment and Independence Support Structures and Program Enhancements Financial and Time Management Support	Self-reliance Flexible program Accountability of supervisors Mentoring program for scholars Workshops for refining the research skills of scholars. Regular feedback from supervisors Financial support for scholars Time management of both supervisors and scholars	"Within the realm of pursuing a doctoral degree, self-reliance plays a crucial role, acting as a guiding principle that aligns with a program designed to foster scholars’ independence." Participant C ”Supervisors play a crucial role in ensuring responsibility and facilitating a collaborative process. This is further enhanced by mentoring programs and research-related workshops promoting personal and professional development. Collectively, these components form a harmonious story, echoing the determined nature of those pursuing a doctoral degree.” Participant F

### Progress, not perfection: Enablers:

Almost all participants identified that scholars need to learn skills regarding academic writing and research skills. The majority of participants recognised that the key factors contributing to the successful progress of a PhD scholar include commitment and punctuality, effective time management, proficiency in academic writing, self-management abilities, guidance from ethics and research committees, as well as support from supervisors, university, and peers. The corresponding codes and representative quotes are outlined in Table-I.

### Confined by the invisible walls: Inhibitors:

Most participants, including scholars and supervisors, stated that PhD scholars often face significant obstacles due to budgetary restrictions and a scarcity of resources. The main obstacles in the successful progression of a PhD scholar were identified as insufficient support and feedback from the supervisor, inadequate support from the university, improper curriculum sequencing, heavy workload and time constraints imposed by supervisors, lackadaisical attitude of scholars towards research, and a lack of dedication and punctuality among scholars. Furthermore, it has been proposed that colleges should establish adaptable programs and effective mentoring initiatives for PhD students to surmount the obstacles they encounter during their academic pursuit. The relevant codes and representative quotes are detailed in Table-I.

### Key to Success: Recommendations:

The participants offered suggestions for the timely completion of the PhD. They suggested financial support as the most important element as lack of finances is the biggest hurdle in their doctoral pathway. Furthermore, they recommended that supervisors should be made accountable for the delay in scholars’ progress. Additionally, it was also suggested that universities should arrange workshops for scholars, especially for research skills. They also recommended that PhD scholars should work on their time management skills and develop self-reliance and self-management competencies to effectively navigate their doctoral pursuits. The relevant codes and representative quotes are detailed in Table-I.

## DISCUSSION

This study offers valuable insights into the experiences of PhD scholars in Pakistan, specifically in medical education, emphasizing the challenges encountered in the absence of institutional support. It integrates insights from scholars and supervisors to reveal under-explored domains, including environmental influences, supervisory dynamics, lack of resources, support systems, and socio-cultural influences on PhD advancement in low—and middle-income countries. Our results depicted the journey of PhD as a very tough undertaking, with every day being a challenge. The theme “*Always alone in the struggle: Experience*” highlights the collective recognition of the daunting obstacles encountered by PhD scholars. Our participants found it was a very tough process, with research work, analysis of data, and thesis writing being the toughest. Pyhältö K et al. highlighted that PhD students found self-regulated learning, maintaining motivation, self-efficacy beliefs, and time management hard.^12^ It was challenging to stand alone and learn to be independent in research work. It required initiative and taking responsibility.^13^ Lindsay et al. reported that PhD research is a challenging endeavour, and writing a doctoral thesis can present one of the most challenging aspects of the PhD journey overall.^14^

Most of the participants had the perspective that marital status, socioeconomic status, and age have significant effects on the journey of a PhD scholar, as married scholars have more responsibilities for their spouses and children. They needed more free time to manage family affairs, while the university required them to attend the faculty full time. Their journey through a PhD was more complicated compared to unmarried scholars.^15^ Factors such as Gender, age, and field of study are variables that influence how PhD researchers negotiate and use their time on their research.^16^

The good socioeconomic status of scholars facilitates their PhD journey as most of the projects undertaken for PhD are not funded. This forms a tremendous burden as the scholar must fund their project and pay hefty university fees on top of earning a livelihood. Phan HP also concluded in a study that financial pressure is one of the factors leading to dropout in PhD candidates.^17^ The primary and most conspicuous theme identified by Beasy K et al. was around apprehensions regarding time and finances, the interaction between financial instability and time constraints experienced by doctorate candidates.^18^ Regarding the theme “*Progress, not perfection: Enablers,”* almost all participants identified that scholars need to learn skills regarding academic writing and research skills. This is integral to the quality of research work and the formulation of a PhD thesis.^19^ Enablers for the successful journey of a PhD scholar reported were dedication, punctuality, and time management. Good time management and self-management skills by PhD students at different universities have been emphasised as vital qualities. Many brilliant students never finalise their thesis because of poor self and time management skills.^14^

Support from supervisors, peers, and the university was highlighted as valuable enablers in the journey to PhD. Supervisory support is vital, for they must provide the expertise and time to foster in the candidate the skills and attitudes of research and to ensure the creation of a thesis that is of an acceptable standard.^20^ It was found in a study by Wollast R et al. that both men and women experience a beneficial impact on their emotional well-being and intention to pursue a PhD route from two aspects of supervisor support: perceived structure and autonomy.^21^

The theme *“Confined by the invisible walls: inhibitors”* highlighted that the main inhibitors in the successful journey of a PhD scholar are the lack of support and feedback from the supervisor. Laufer M et al. have highlighted the hierarchical power dynamics between the supervisors and PhD scholars, especially in the context of feedback experiences with students suffering consequences for challenging their supervisor’s authority or not meeting expectations.^4^ The supervisors, on the other hand, bemoaned the dearth of dedication and motivation among their trainees, as shown by their lack of punctuality. Stubb et al. showed that more than half of the PhD in the field of medicine found their relationships with their scholarly communities burdening.^22^ Thus, the reported high attrition rates among PhD students, the dropout rate ranges from 30 to 50% - depending on the discipline and country.^4^

The theme *“Key to Success: recommendations”* notes that the participants felt the need for financial support. Their perspective is supported by much of the literature.^22^ Participants highlighted the need for scholars to work on their time and self-management and develop self-reliance to complete this crucial journey as scholars. Although hard to develop, these are vital to successfully achieving a PhD.^14^ Moreover, it was also suggested that workshops for refining the research skills of scholars should be arranged.

This study emphasizes the challenges faced by PhD scholars in medical sciences, who play a crucial role in healthcare education and research. Isolation, strained relationships between supervisors and scholars, and workload pressures adversely affect the effectiveness of teaching and research activities. The study highlights the importance of customized support systems to mitigate burnout, improve work-life balance, and promote a healthy academic environment.

### Strengths

This study’s strength is its dual perspectives, offering a comprehensive understanding by including scholars and supervisors. This study represents one of the initial investigations into PhD scholars in medical education within Pakistan, thereby addressing a significant gap in the regional literature. The qualitative approach facilitated a rigorous exploration of complex experiences and key emerging themes.

### Limitations:

Our study is susceptible to numerous limitations. The results are indicative of the cultural and socio-economic context of Pakistan, which may preclude their generalizability to PhD scholars in other cultural contexts, particularly in non-developing countries. This study offers an overview of PhD scholars’ experiences but fails to document fluctuations in their experience over time. A longitudinal approach could have elucidated the evolution of coping mechanisms during the PhD journey.

## CONCLUSION

The journey of a PhD is a very tough undertaking, with every day being a challenge. It requires initiative and taking responsibility. Marital and socioeconomic status have significant effects on their journey. The dearth of funded projects means scholars must juggle full-time jobs along with their PhD tasks and responsibilities. Lack of support and feedback from the supervisor, along with financial constraints, are major barriers for PhD scholars. Dedication, punctuality, and time management are necessary to develop good academic writing and research skills. Support from supervisors, peers, and the university serves as valuable enablers.

### Future direction:

Future research should explore comparative studies across various disciplines and institutions to determine effective practices for supporting PhD scholars. Research focused on interventions is necessary to evaluate the effects of support programs on scholars’ academic performance and mental health. Longitudinal studies examining the academic progression of scholars over time would yield significant insights into their long-term challenges and outcomes.
